# Surgery of secondary mitral insufficiency in patients with impaired left ventricular function

**DOI:** 10.1186/1749-8090-4-36

**Published:** 2009-07-17

**Authors:** Andreas Rukosujew, Stefan Klotz, Henryk Welp, Christian Bruch, Farshad Ghezelbash, Christoph Schmidt, Raluca Weber, Andreas Hoffmeier, Jürgen Sindermann, Hans H Scheld

**Affiliations:** 1 Department of Thoracic and Cardiovascular Surgery, University Hospital Muenster, Germany; 2 Department of Anesthesiology and Operative Intensive Care Medicine, University Hospital Muenster, Germany; 3 Department of Cardiology and Angiology, University Hospital of Muenster, Germany

## Abstract

**Background:**

Secondary mitral insufficiency (SMI) is an indicator of a poor prognosis in patients with ischemic and dilated cardiomyopathies. Numerous studies corroborated that mitral valve (MV) surgery improves survival and may be an alternative to heart transplantation in this group of patients.

The aim of the study was to retrospectively analyze the early and mid-term clinical results after MV repair resp. replacement in patients with moderate-severe to severe SMI and left ventricular ejection fraction (LVEF) below 35%.

**Methods:**

We investigated 40 patients with poor LVEF (mean, 28 ± 5%) and SMI who underwent MV repair (n = 26) resp. replacement (n = 14) at the University Hospital Muenster from January 1994 to December 2005. All patients were on maximized heart failure medication. 6 pts. had prior coronary artery bypass grafts (CABG). Twenty-seven patients were in New York Heart Association (NYHA) class III and 13 were in class IV. Eight patients were initially considered for transplantation. During the operation, 14 pts had CABG for incidental disease and 8 had tricuspid valve repair. Follow-up included echocardiography, ECG, and physician's examination and was completed in 90% among survivors. Additionally, the late results were compared with the survival after orthotope heart transplantation (oHTX) in adults with ischemic or dilated cardiomyopathies matched to the same age and time period (148 patients).

**Results:**

Three operative deaths (7.5%) occurred as a result of left ventricular failure in one and multiorgan failure in two patients. There were 14 late deaths, 2 to 67 months after MV procedure. Progress of heart failure was the main cause of death. 18 patients who were still alive took part on the follow-up examination. At a mean follow-up of 50 ± 34 (2–112) months the NYHA class improved significantly from 3.2 ± 0.5 to 2.2 ± 0.4 (p < 0.001). The LVEF improved significantly from 29 ± 5% to 39 ± 16 (p < 0.05). There were no differences in survival after MV repair or replacement. The 1-, 3-, 5-year survival rates in the study group were 80%, 58% and 55% respectively. In the group of patients after oHTX the survival was accordingly 72%, 68%, 66% (p > 0.05).

**Conclusion:**

High risk mitral valve surgery in patients with cardiomyopathy and SMI offers a real mid-term alternative method of treatment of patients in drug refractory heart failure with similar survival in comparison to heart transplantation.

## 

Mitral valve insufficiency associated with a considerably impaired left ventricular (LV) function is not a homogeneous clinical entity. On the one hand, it could be a component of the mitral valve disease itself as a primary insufficiency. On the other hand, it could be secondary or functional as a manifestation of a late stage of different forms of heart pathology such as dilated cardiomyopathy and ischemic heart disease. In patients with end-stage cardiomyopathy secondary mitral insufficiency (SMI) occurs in approximately 60% and is associated with a poor prognosis [1-3]. Myocardial damage by infarction or from dilated cardiomyopathy leads to leakage of the anatomically normal MV. Following dilatation of the annular-ventricular apparatus, papillary muscle displacement, and an altered ventricular geometry, left ventricular volume overload occurs which decreases leaflet coaptation and worsened mitral regurgitation [4,5]. Due to increased risk for perioperative death and presumably worse long-term results some authors recommended conservative treatment in patients with SMI and severe left ventricular dysfunction [6]. In a review of this cohort 1-year mortality after diagnosis of SMI has been reported between 34% and 70% [7-10]. It has been shown that 50% of these patients die within 3 years after first admission to the hospital without surgical treatment [10].

For cardiomyopathy with SMI, heart transplantation was the treatment of choice in most institutions, since mitral valve surgery in patients with severe heart failure had been identified as an indicator of poor prognosis in numerous studies [11-13]. Although, cardiac transplantation has encouraging results for these patients with SMI and end-stage heart failure, transplantation is hindered by donor organ shortage and its limited applicability to older patients or those with concurrent diseases.

Along with the increasing shortage of appropriate donor organs and improved surgical techniques, various concepts of high risk mitral valve surgery evolved. Studies could show improved exercise capacity following mitral valve operation in these patients [14,15]. It was shown that end-diastolic and end-systolic dimensions and volumes were lessened and stroke volume and ejection fraction increased [3,16,17]. We have previously reported on our experience with high risk mitral valve surgery -in patients with severe left ventricular dysfunction [18]. The aim of this paper is to analyze the mid-term outcome and the cause of late death in this patient group. Furthermore we evaluated from our late results if MV repair or replacement have influence on survival. In addition we compared these results with the late results after heart transplantation in an age, gender and time-period matched patient group.

## Patients and methods

From January 1994 to December 2005, 40 consecutive patients with SMI (grade III-IV) and impaired left ventricular pump function (EF < 35% in echocardiography and angiography) underwent mitral valve surgery at our institution. Patients with previous coronary artery bypass grafting (CABG) were included in the study; patients with an additional left ventricular remodeling procedure were excluded.

The study population included 20 male and 20 female patients with a mean age of 64 ± 9 years. 26 patients suffered from dilative cardiomyopathy, 14 from ischemic cardiomyopathy. Mean preoperative ejection fraction was 28 ± 5%. 24 had chronic atrial fibrillation. 27 patients were in New York Heart Association (NYHA) class III and 13 patients in NYHA class IV despite optimized heart failure therapy which typically included digoxin, angiotensin – converting enzyme inhibitors, diuretics, beta – blockers and spironolactone. 16 patients had been initially referred to our institution for evaluation of heart transplantation. Patient characteristics and comorbidities are shown in Table 1.

**Table 1:** Patient characteristics and comorbidity

Variable	
Sex (f/m)	20/20
Cardiomyopathy idiopathic/ischemic	26/14
Age (years)	64 ± 9
NYHA class	3.3 ± 0.5
Atrial fibrillation	24 (60%)
Creatinine > 1.5 mg/dl	27 (67.5%)
History of stroke	6 (15%)

Data presented as mean ± SD and total number

NYHA = New York Heart Association

All patients underwent transthoracic or transoesophageal echocardiography on hospital admission, transoesophageal echocardiography after induction of anesthesia prior to sternotomy and intraoperative to control the results after mitral valve surgery. Postoperative follow-up echocardiography was obtained prior to discharge and on follow-up. If no follow-up was available in our institution (two patients), the investigation was performed at a city hospital.

The perioperative measurement of left ventricular chamber size at end-diastole and end-systole as well as the assessment of mitral regurgitation were performed by transthoracic two-dimensional echocardiographic images in the parasternal long-axis view (including M-mode) and by apical four-chamber view. The intraoperative analysis was performed by employing short-axis and long-axis views using a transoesophageal approach. Left ventricular volumes and ejection fraction were calculated by modification of Simpson's rule method with two apical views. Stroke volume was calculated as the difference between the diastolic and systolic volumes, and ejection fraction was calculated as the ratio of stroke volume to end-diastolic volume. Colour Doppler flow mapping of regurgitant jets with visualisation of the vena contracta and proximal isovelocity surface area was used for quantification of valvular regurgitation. The severity was graded as mild (I), moderate (II), moderate-severe (III), and severe (IV) [3,19].

29 patients (72.5%) had grade 3, and 11 (27.5%), grade 4 mitral regurgitation. All patients had normal or moderate impaired right ventricular function without presence of liver failure, although in 10 patients the mean pulmonary pressure was higher than 40 mm Hg.

Preoperative echocardiography and hemodynamic data are listed in Table 2.

**Table 2:** Echocardiography and right heart catheter data

Variable	
LVEDD (mm)	65 ± 7
LVESD (mm)	47 ± 8
LVEDV (ml)	191 ± 67
LVESV (ml)	118 ± 53
LVEDP (mmHg)	23 ± 8
SV (ml)	53 ± 17
LVEF (%)	28 ± 5
V – Wave (mmHg)	43 ± 14
Pulmonary artery systolic pressure (mmHg)	55 ± 14
Pulmonary artery diastolic pressure (mmHg)	26 ± 9
Mean pulmonary artery pressure (mmHg)	35 ± 11
Pulmonary capillary wedge pressure (mmHg)	23 ± 6
Mitral regurgitation (grade)	3.2 ± 0.4
Cardiac index (L/min/m^2^)	2.0 ± 0.3

Data presented as mean ± SD

LVEDD = left ventricular end-diastolic diameter; LVESD = left ventricular end-systolic diameter; LVEDV = left ventricular end-diastolic volume; LVESV = left ventricular end-systolic volume; LVEDP = left ventricular end-diastolic pressure; SV = stroke volume; LVEF = left ventricular ejection fraction.

### Surgical Procedure

Mitral valve surgery was performed through a median sternotomy, establishing a cardiopulmonary bypass with a moderate systemic hypothermia. Myocardial protection was administered using an intermittent retrograde cold blood cardioplegia and topical cooling in all patients. The operative data are outlined in Table 3. The MV was exposed through the interatrial septum only or through left atrial roof and interatrial septum. 26 patients underwent mitral valve repair, 12 of whom had a quadrangular resection of the posterior leaflet (P2), and six had commissural plasties according to Whooler (n = 2) or Kay (n = 4). No chordal transfer or Alferi stitch was performed in this patient group. In all patients, a moderately undersized Carpentier Edwards classic annuloplasty ring (28 – 30 mm) was inserted as a part of the repair, even after Kay or Whooler commissural plasties [20].

**Table 3:** Operative data

Variable		
OP Duration		181 ± 46
X-clamp (minutes)		51 ± 23
CPB time (minutes)		111 ± 40
Mitral valve repair		26
Isolated	14	
+ Tricuspid valve repair	4	
+ CABG	7	
+ Tricuspid valve repair + CABG	1	
Mitral valve replacement		14
Isolated	5	
+ Tricuspid valve repair	3	
+ CABG	6	
Bioprosthesis		9
Mechanical valve		5

Data presented as mean ± SD and total number

X – clamp = cross-clamping time; CPB = cardiopulmonary bypass; CABG = coronary artery bypass grafting

Mitral valve replacement was unavoidable in 14 patients. A Carpentier Edwards bioprosthesis was implanted in nine patients, and a mechanical St. Jude Medical valve in five patients. All efforts were made to preserve the posterior leaflet with the subvalvular apparatus.

Fourteen patients underwent a combined procedure with additional coronary artery bypass grafting (CABG) which was performed first, using the mammary artery in all cases and if necessary a saphenous vein graft (mean, 2 ± 1 distal anastomoses).

An additional tricuspid valve annuloplasty was performed in 8 patients after MV surgery. A De Vega procedure was used in 6 patients and an annuloplasty ring in 2 patients.

### Follow-up

In 34 (92%) from 37 patients, who were discharged from the hospital after operation, mid-term results were analyzed. 14 patients died during follow-up. 20 patients were still alive and complete follow-up was available. All patients were interviewed by telephone, and were invited to an examination on an ambulatory basis. 18 patients agreed to participate on the follow-up examination at our institution. Two patients underwent follow-up examination at other institutions.

### Statistics

The paired Student *t*-test was used to compare groups. The log rank test was used in the Kaplan-Meier Survival analysis. Data are expressed as mean ± SD. A *p*-value of less than 0.05 was considered statistically significant.

## Results

### Operative mortality and morbidity

Postoperative data with duration of mechanical ventilation, ICU treatment, complications and hospital stay are listed in Table 4. All patients required moderate or high dose inotropic treatment to wean from cardiopulmonary bypass. In ten patients (25%) an intraaortic balloon pump (IABP) was inserted, in 9 patients intraoperatively to facilitate termination of cardiopulmonary bypass and in one for perioperative myocardial infarction.

**Table 4:** Postoperative data

Variable	
Death	3 (7.5%)
AMI	1 (2.5%)
Stroke	0
IABP	10 (25%)
Dialysis	3 (7.5%)
Reintubation	3 (7.5%)
Postoperative bleeding (mL)	854 ± 618
Redo for bleeding	2 (5%)
Transient psychosis	4 (10%)
Ventilation (hours)	16.9 ± 14.9
ICU stay (days)	2.5 ± 4.7
Ventrical tachycardia	2 (5%)
Sternal infection	1 (2.5%)
Hospital stay (days)	8.8 ± 4.7

Data presented as mean ± SD and total number

AMI = acute myocardial infarction; IABP = intraaortic balloon pump; ICU = intensive care unit.

Three patients (7.5%) died within thirty days after surgery. One patient died 1 day after MV replacement and CABG following acute myocardial infarction. One death occurred 10 days postoperatively as a consequence of severe right ventricular dysfunction and secondary multi-organ failure. The other patient died in a septic shock on day 28. Mean intensive care unit stay was 2.5 ± 4.7 days. Mean hospital stay was 8.8 ± 4.7 days.

Two patients (5%) required operative re-exploration because of bleeding.

Three patients needed readmission in the ICU for acute respiratory insufficiency. Furthermore, three patients with preoperative renal impairment needed dialysis postoperatively for acute renal failure. In 2 patients (5%) an ICD was implanted due to ventricular tachycardia.

### Follow-up results

Follow-up data were completed in March 2007. There were 14 late deaths during the follow-up period, 2 to 67 month after MV procedure (Table 5). Eight patients died related to cardiac reasons (heart failure = 7, sudden death = 1). There were no differences in late mortality for ischemic or dilated cardiomyopathy in these 8 patients. The other five patients died from not heart failure-related causes: pneumonia with sepsis in two cases, stroke, cerebral bleeding and cancer complicated with intestine perforation in one patient. In one patient the cause of death was unknown. After a follow-up period of 50 ± 34 months (range, 2–112 month), mean NYHA functional class significantly improved from 3.3 ± 0.5 at operation to 2.2 ± 0.4 (p < 0.001). There were no differences between ischemic or dilated cardiomyopathy patients. A late echocardiographic study (62 ± 29 months) was available in 18 cases and only echocardiographic examinations performed at our institution were included (Table 6). The left ventricular ejection fraction had increased from 29 ± 4% to 39 ± 16% at follow-up (p < 0.05). The mitral valve regurgitation severity decreased significant from Grade 3.2 ± 0.4 to moderate regurgitation Grade 1.5 ± 0.4 at follow-up (p < 0.001). The left ventricular end-diastolic diameter decreased from 64 ± 5 to 60 ± 7 mm (p = 0.062) and the echocardiographic parameters of systolic pulmonary artery pressure and pulmonary capillary wedge pressure show a significant reduction of pulmonary hypertension (p < 0.01).

**Table 5:** The cause of late death

Mortality	No of pts
Total deaths (2 to 67 months)	14
Progress of heart failure (3,4,6,16,31,34,56,67 months)	7
Sudden death (9 months)	1
Pneumonia with sepsis (25 and 27 months)	2
Stroke (26 months)	1
Intestine perforation, peritonitis (2 months)	1
Cerebral bleeding (17 months)	1
Unknown (30 months)	1

**Table 6:** Follow-up echocardiographic and clinical examinations

Variable	preoperative n = 18	postoperative n = 18	*p *Value
LVEDD (mm)	64 ± 5	60 ± 7	0.062
LVEDV (ml)	185 ± 37	166 ± 61	0.298
LVEDP (mmHg)	25 ± 6	19 ± 5	0.122
EF (%)	29 ± 4	39 ± 16	0.038
PAs (mmHg)	58 ± 13	41 ± 9	0.001
PCWP (mmHg)	26 ± 6	17 ± 5	0.007
Mitral regurgitation (grade)*	3.3 ± 0.4	1.5 ± 0.4	0.001
Cardiac index (L/min/m^2^)	2.1 ± 0.3	2.4 ± 0.5	0.195
NYHA	3.2 ± 0.5	2.2 ± 0.4	0.001

Data presented as mean ± SD

*Only in patients with mitral valve repair (n = 13).

**Figure 1:**
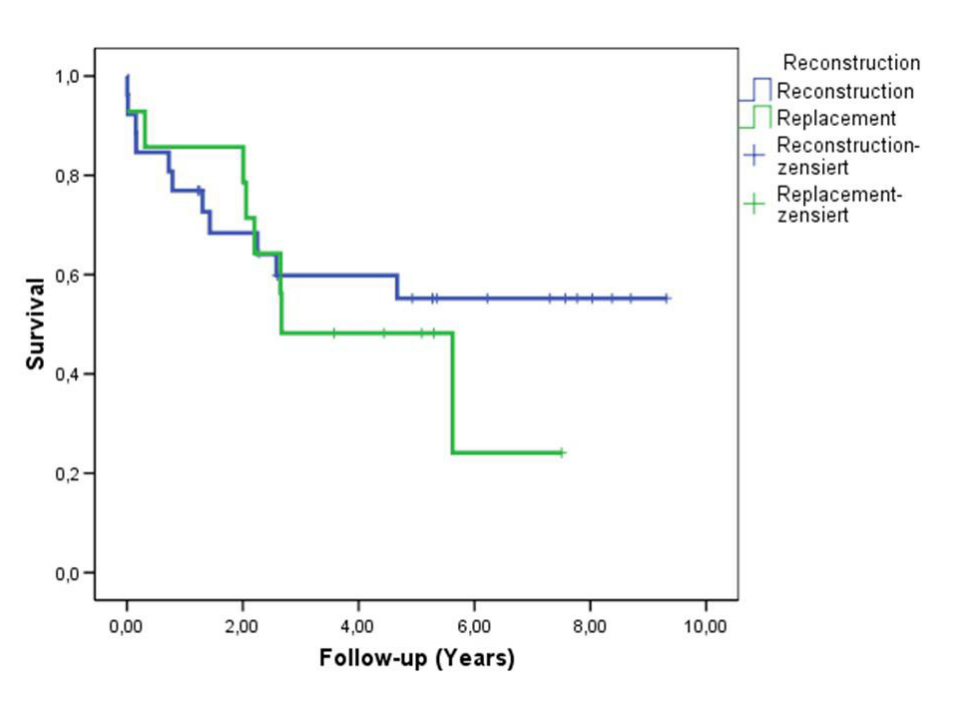
**Kaplan – Meier survival curve in patients after MV reconstruction and MV replacement**. Log Rank 0.541.

**Figure 2:**
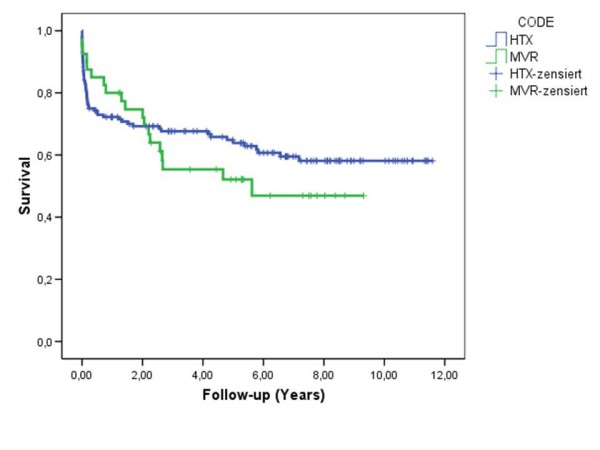
**Kaplan – Meier survival curve in patients after MV surgery (MVR) and heart transplantation (HTX)**. Log Rank 0.426.

LVEDD = left ventricular end-diastolic diameter; LVEDV = left ventricular end-diastolic volume; LVEDP = left ventricular end-diastolic pressure; LVEF = left ventricular ejection fraction; PAs = systolic pulmonary artery pressure;. PCWP = pulmonary capillary wedge pressure; NYHA = New York Heart Association:

The 1-, 3-, 5-year survival rates in the study group were 80%, 58% and 55% respectively. There were no differences in survival after MV repair or replacement (Fig. 1). Additionally we have compared the late results in the study group with the survival after cardiac transplantation in 148 age, gender and time-period matched patients with dilated and ischemic cardiomyopathy (Fig. 2). In this group the survival was accordingly 72%, 68%, 66% and similar to patients after MV surgery (p > 0.05).

From ten patients with preoperative pulmonary hypertension with mean pulmonary pressure higher 40 mmHg three patients died owing to progressive heart failure, six were still alive at time of follow-up examination (range 53 – 100 month) and in one patient was lost to follow-up.

## Discussion

Surgical treatment in patients with SMI and considerably impaired LV function had been identified as an indicator of poor prognosis. This is due to the primary ventricular problem that causes MV dysfunction and, despite of the SMI correction, the disease further exists [21]. MV surgery is associated with a high mortality which in the series of other investigators varies between 2.3% and 19.4% [3,16,22]. The patients in our study, therefore, represented a high predicted mortality among patients with dilated or ischemic cardiomyopathy. The surgical mortality in patients with ischemic cardiomyopathy with an age > 60 years have been reported from 10 to 48% [23]. A high operative mortality of 21% has been reported among a group of 28 patients undergoing mitral valve replacement and additional CABG [24]. Despite high operative risks the 30-days mortality in our group of patients was moderate with 7.5% (n = 3). All patients with early death had ischemic cardiomyopathy with previous CABG. In addition, in two cases it was a combination of MV replacement with CABG and in one a combination of MV repair, CABG and TV repair. Redo's and prolonged cross-clamping times are the mainly responsible for the high mortality in patients with -ischemic cardiomyopathy. There were no deaths in patients with dilated cardiomyopathy and our results confirm the reports of other authors about poor early outcomes in patients with ischemic cardiomyopathy.

MV surgery in patients with LV dysfunction is associated with higher postoperative complication rates. Two thirds of our patients had preoperative atrial fibrillation and/or compensated renal insufficiency and ten of them were older than 70 years. Postoperative morbidity rate exceeded 40% and was expected in this cohort of patients with serious comorbidities. Early complications such as redo for bleeding, acute respiratory failure, acute renal failure with dialysis and ventricular tachycardia developed in 11 (27.5%) of our patients. This is the reason for longer ventilation, ICU- and hospital stay and that coincides with reports of other investigators [3,25].

All patients showed improvement in exercise tolerance at the follow-up, and the NYHA class improved significantly. There were no differences between patients with ischemic or dilated cardiomyopathy. Quantitative echocardiographic analyses showed a markedly increase of ejection fraction, a significant reduction of pulmonary hypertension and of the left ventricular end-diastolic diameter at follow-up. Furthermore six from ten patients (60%) with severe pulmonary hypertension were still alive (mean survival 58 ± 29 months) at time of follow-up. These results do not confirm the opinion of other investigator about poor outcomes in this cohort of patients [26]. The mitral regurgitation improved significantly from severe to moderate at follow-up in the repair/annuloplasty group. The mid-term survival in this study group was none significantly shorter compared to heart transplantation.

Following the guidelines for the management of patients with valvular heart disease asymptomatic patients with an ejection fraction below 60% and/or end-systolic dimension over 40 mm and symptomatic patients with EF above 30% and LVESD below 55 mm are candidates for mitral surgery [27]. Still controversy exists regarding the operation (MVR versus HTX) in patients with severe reduced ejection fraction.

Our results could show that MV surgery in patients with considerably impaired LV function has a similar mid-term outcome compared to transplantation. We therefore conclude that in MV surgery is an acceptable alternative procedure which improved quality of life in the present time with increasing organ shortage.

Of importance is the preservation of the mitral valve apparatus. Previous clinical studies have compared the results of MV reconstruction against those following MV replacement and have concluded that preservation of the annular-chordal-papillary muscle continuity results in maintenance of LV function and geometry, leading to better patient outcome [28-30]. However, we could not observe a difference in outcome between MV repair and replacement. One reason could be the preservation of the mitral valve apparatus despite MV replacement, which was observed in our studies, too [31]. But we think that chordal sparing mitral valve replacement is not a better way to treat SMI because of the need for anticoagulation for mechanical prosthesis in mitral position and inevitable degeneration of bioprosthesis.

Through the restoration of the mitral competency and ventricular geometry, MV surgery offers a new treatment strategy for the treatment of end-stage heart failure.

## Limitations of the study

This study has several limitations. First, the population represents a relatively small number of patients. However, significant differences were detected. Second, the accuracy of the volume quantification is dependent to operator experience, and calculation of flows has inherent errors because of limitations in measurement accuracy. Third, the assessment for the functional status at the follow-up was subjective. We tried to objective our results using standardized exercise tests. Forth, the comparison of the mitral valve with the transplant patient group is not very accurate, because of the more depressed LV function and co-morbidities in the transplant group. However, we tried to overcome this obstacle by age, gender and time period matching.

## Conclusion

High risk mitral valve surgery for secondary mitral regurgitation in patient with ischemic and dilated cardiomyopathies and considerably impaired LV function corrects effectively mitral regurgitation and represents an alternative procedure in a high-risk population with an acceptable perioperative mortality rate. Decrease in mitral regurgitation after surgical correction contributes to restoration of left ventricular geometry and may be an alternative to heart transplantation in selected patients.
